# Water Soluble Thermoresponsive Single‐Chain Cyclized/Knotted Polymers from Intramolecular Cyclization Dominated Homopolymerization of PEG Diacrylate

**DOI:** 10.1002/marc.202401068

**Published:** 2025-03-10

**Authors:** Zishan Li, Jing Lyu, Yinghao Li, Rijian Song, Chunyu Zhao, Melissa Johnson, Tianyu Mao, Hongyun Tai, Wenxin Wang

**Affiliations:** ^1^ Charles Institute of Dermatology School of Medicine University College Dublin Dublin 4 Dublin D04 V1W8 Ireland; ^2^ School of Mechanical and Materials Engineering University College Dublin Dublin 4 Dublin D04 V1W8 Ireland; ^3^ Blafar Limited Cherrywood Business Park, Cherrywood, Loughlinstown, Dublin 18 Dublin D18 T3Y1 Ireland

**Keywords:** Cu(0)&Cu^II^‐mediated RDRP, intramolecular cyclization, LCST, single‐chain cyclized/knotted polymers

## Abstract

A series of water‐soluble, PEG‐based single‐chain cyclized/knotted polymers are successfully synthesized through the homopolymerization of poly(ethylene glycol) diacrylate (PEGDA) (*M*_n_  = 575 and 700 g mol^−1^, respectively) via enhanced intramolecular cyclization. The homopolymerization of the diacrylate macromers first proceeds through linear propagation, followed by self‐cyclization. Under high monomer‐to‐initiator ratios (e.g., 100–500) and room temperature, cyclized polymers with high cyclized ratios of up to 70% are achieved without gelation. Notably, the cyclized poly(PEGDA_575_) exhibits concentration‐dependent thermoresponsive properties in aqueous solutions.

## Graphical abstract

A series of water‐soluble, PEG‐based single‐chain cyclized/knotted polymers are successfully synthesized via the homopolymerization of PEGDA (*M*_n_ = 575 and 700 g mol⁻¹, respectively), facilitated by enhanced intramolecular cyclization. These cyclized polymers feature a high cyclization ratio and possess numerous pendant vinyl groups, providing excellent versatility for post‐functionalization. Notably, the cyclized poly(PEGDA_575_) demonstrates concentration‐dependent thermoresponsive behavior in aqueous solutions.

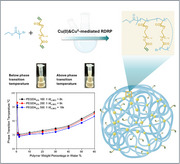

## 1 Introduction

Although synthetic polymers can exhibit a variety of topologies, such as linear, branched, hyperbranched, star‐shaped and brush‐like, polymers composed of cyclic units (macrocyclic,^[^ ^1^ ^]^ multicyclic,^[^ ^2^, ^3^, ^4^ ^]^ knotted cyclic,^[^ ^5^, ^6^, ^7^ ^]^ and folded cyclic^[^ ^8^, ^9^, ^10^ ^]^) have garnered particular attention due to their compact architectures and distinctive properties. These properties include high density, low intrinsic viscosity, reduced translational friction, elevated glass transition temperatures,^[^ ^11^ ^]^ enhanced network elasticity,^[^ ^12^ ^]^ lower cytotoxicity, and prolonged circulation times in drug and gene delivery applications.^[^ ^13^, ^14^, ^15^ ^]^ However, the efficient and practical synthesis of cyclic architecture remains a significant challenge for polymer chemists, as it requires polymer chains to undergo intramolecular reactions before reacting with other chains. To facilitate these intramolecular reactions, approaches typically involve working under highly dilute conditions,^[^ ^16^ ^]^ utilizing 1D channels,^[^ ^17^ ^]^ or employing cyclopolymerization of specifically designed monomers that favor intramolecular reactions through steric control and energy lowering effect.^[^ ^18^, ^19^ ^]^

Interestingly, a Cu(0)&Cu^II^‐mediated reversible‐deactivation radical polymerization (RDRP) method was previously reported as an effective strategy for controlling the homopolymerization of multifunctional vinyl monomers (MVMs).^[^ ^20^ ^]^ This kinetically controlled approach has demonstrated that it can regulate both the gelation point and the macromolecular architecture in MVM homopolymerization, without requiring dilute reaction conditions. This method successfully postponed the gelation point to higher than 40% monomer conversion (*Conv*_._).^[^ ^20^, ^21^ ^]^ Moreover, a novel polymer architecture was formed, primarily driven by intramolecular knotting within a single polymer chain, rather than the typical hyperbranching through intermolecular cross‐linking. This newly discovered structure, where individual polymer chains fold back and knot upon themselves, is referred to as a “single cyclized” molecular structure, in contrast to the “single branched” structure characteristic of dendrimers.^[^ ^16^, ^20^, ^22^ ^]^

**Scheme 1:**
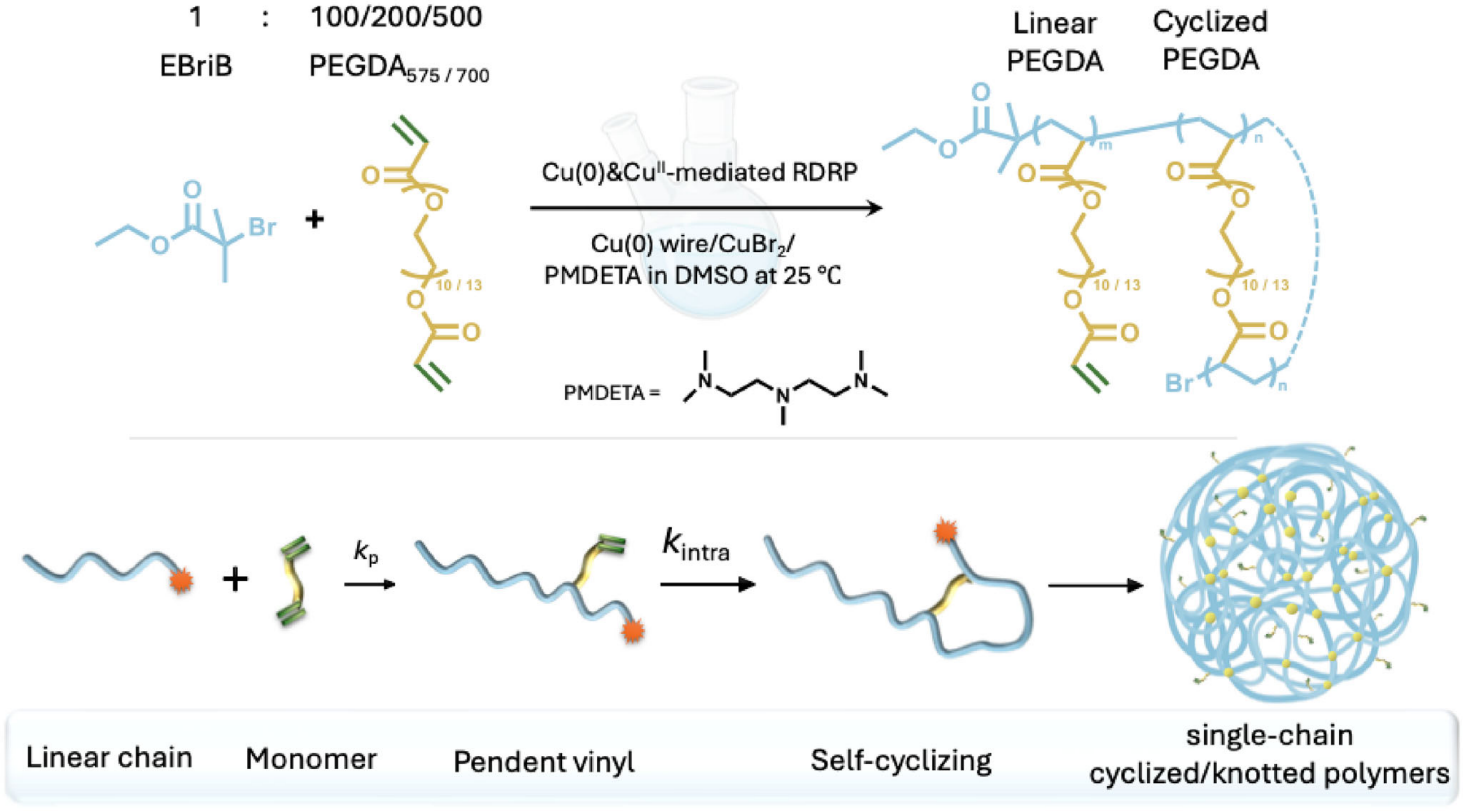
Homopolymerization of PEGDA_575_ and PEGDA_700_ and formation of single‐chain cyclized/knotted polymers through intramolecular cyclization. The yellow circles in the schematic representation represent the intramolecular cyclization linkages, formed by the reactions of active radicals and pendent vinyl groups on the same chain.

In this study, two poly(ethylene glycol) diacrylates (PEGDA), PEGDA_575_ (average *M*_n_ = 575) and PEGDA_700_ (average *M*_n_ = 700), with different PEG spacer lengths (n = 10 and 13, respectively, as depicted in **Scheme** **1**), were homopolymerized using Cu(0)&Cu^II^‐mediated RDRP. This process resulted in a series of water‐soluble polymers with single cyclized/knotted structures. The propensities of intramolecular cyclization were examined using both size exclusion chromatography (SEC) and proton nuclear magnetic resonance (^1^H NMR) analysis. Additionally, the concentration‐dependent phase transition behavior of the synthesized poly(PEGDA_575_) was investigated.

## 2 Results and Discussion

Successful control of the homopolymerization of MVMs relies on effectively managing chain propagation, intramolecular cyclization, and intermolecular cross‐linking processes. This requires maintaining a minimal active kinetic chain length (AKCL), or growth boundary, which restricts the number of vinyl groups that can react with the propagation center during its active lifetime.^[^ ^23^ ^]^ Based on our previous research, the strategy for achieving a small growth boundary (AKCL) in Cu‐catalyzed polymerization systems is enhancing the deactivation strength, and regarding the type of acrylates monomers (which has a high chain propagation rate constant), increasing the amount of deactivator (Cu^II^) is the most straightforward and efficient method that can be adapted.^[^ ^24^ ^]^ Therefore, for PEGDA in this work, extra deactivator Cu^II^ was introduced into the system. Under small AKCL control (AKCL = 0.15 for all polymerization systems in this work), to promote intramolecular cyclization over intermolecular cross‐linking, a high molar ratio of divinyl monomer to initiator was used – 500:1, 200:1, and 100:1 – for both PEGDA_575_ and PEGDA_700_. The reaction conditions, along with the characterization data of the polymer products at different times, are detailed in **Table** **1** and Table S1 (Supporting Information).

**Table 1:** Reaction results for the homopolymerization of PEGDA_575_ and PEGDA_700_ via Cu(0)&Cu^II^‐mediated RDRP.

	Diacrylate	M: I^a)^	Time [h]	*Conv*_._ ^b)^ (%)	*M*_n,SEC_ ^c)^ [kDa]	*M*_w,SEC_ ^c)^ [kDa]	*Đ* ^c)^	*Vinyl Content* ^d)^	Cyclized ratio.^d)^ (%)	Initiator content^d)^ (%)	*M*_n,NMR_ ^d)^ [kDa]	*α* ^e)^
1	PEGDA_700_	100:1	2.5	17.4	7.57	10.46	1.32	39.7	60.3	6.72	10.42	0.26
2		200:1	4	16.4	11.14	13.47	1.21	44.6	55.4	3.52	19.89	0.36
3		500:1	6	11.4	22.04	25.67	1.17	49.0	51.0	1.55	45.16	0.31
4	PEGDA_575_	100:1	2.5	16.1	6.65	7.98	1.20	28.4	71.6	7.35	7.82	0.32
5		200:1	3	13.5	7.69	9.00	1.17	30.9	69.1	3.34	17.46	0.28
6		500:1	5	10.7	15.13	17.15	1.14	41.8	58.2	1.93	29.79	0.34

^a)^ [M]_0_/[I]_0_/[Cu^II^]_0_/[L]_0_ = 100/[I]_0_(= 1; 0.5; 0.2)/0.4/0.8; M: polyethylene glycol diacrylate; I: ethyl α‐bromoisobutyrate (EBriB), Cu^0^ = pretreated Cu(0) wire (*l* = 5 cm, *d* = 1 mm); Cu^II^ = CuBr_2_, L = PMDETA; solvent = DMSO;

^b)^ The monomer conversion is calculated by comparing the integrated areas of the polymer and monomer peaks in the SEC trace;

^c)^ *M*_n,SEC_, *M*_w,SEC_ and *Đ* are determined by SEC using PMMA as standards in DMF;

^d)^ Calculated by ^1^H NMR as seen in Figure 2 and Equations S1–S4 (Supporting Information). The Cyclized ratio is equal to the pendent vinyl conversion before intermolecular combinations happen;

^e)^ Mark–Houwink exponent *α*.

**Figure 1:**
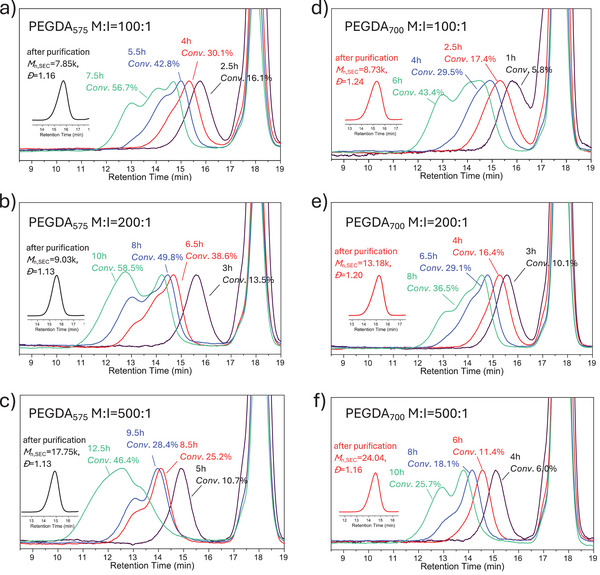
SEC traces of the polymerization mixtures taken at different reaction times for the homopolymerizations of both PEGDA_575_ and PEGDA_700_ at different initiator‐to‐monomer ratios.

As shown in **Figure** **1**, the gelation points of six polymerization systems ([M]/[I] is from 100/1 to 500/1) were all significantly delayed, with no gelation observed after 6.5 to 12.5 h, during which *Conv*. reached 51.3% to 78.1% from 500/1 system to 100/1 system (Table S1, Supporting Information). This observation contradicts the conventional understanding of MVM polymerization, as these *Conv*. values are notably higher than those predicted by the Flory–Stockmayer theory (should be lower than 6% to 14% from 500/1 system to 100/1 system).^[^ ^25^, ^26^, ^27^, ^28^ ^]^ Basically, the polymerizations in Figure 1 show two stages. In stage 1 (before the appearance of multimodal SEC peaks), a clear linear correlation was observed between molecular weight and monomer conversion (Figure S1, Supporting Information). During this stage, the dispersity (*Đ*) remained low (*Đ* < 1.4), and the SEC traces exhibited unimodal peaks. This behavior sharply contrasts with hyperbranched polymerization systems, where rapid chain coupling often results in a significant increase in molecular weight accompanied by a *Đ* value far greater than 1.5 with multimodal GPC.

In addition, the values of *M*_n,NMR_ were in good agreement with the theoretical value (*M*_n,th_), which further proved the controlled linear chain feature at stage 1 (some underestimated molecular weights were observed in *M*_n,SEC_ compared to *M*_n,th_ due to the use of linear PMMA as SEC calibration standards. Cyclized/knotted polymers generally exhibit a smaller hydrodynamic volume compared to linear polymers of the same molecular weight. As SEC separates molecules based on their hydrodynamic volume, this structural difference led to an underestimation of the molecular weight for cyclized or compact structures). Therefore, it can be concluded that polymer chains grew linearly without intermolecular reactions occurring during this initial stage, leading to monodisperse primary chains without chain coupling. In stage 2 of the polymerization, once the polymer concentration surpassed certain concentration (critical overlap concentration),^[^ ^29^ ^]^ the polymer chains began to undergo inevitable combination, as evidenced by a sharp increase in molecular weight with a *Đ* value above 1.5, along with multimodal SEC peaks (Figure S1, Table S1, Supporting Information).

**Figure 2:**
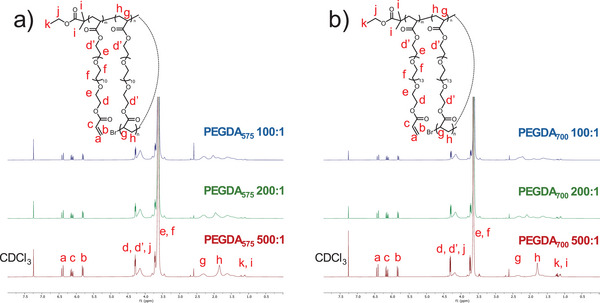
^1^H NMR spectra of purified polymers (from Table 1) obtained by homopolymerization of a) PEGDA_575_ and b) PEGDA_700_ with different monomer‐to‐initiator ratios.

Furthermore, the conversion of pendent vinyl groups (Cyclized ratio in Table 1) in the samples taken from the linear growth stage was found to be unexpectedly high (51% with 11.14% *Conv*., and 58.2% with 10.7% *Conv*. in Table 1, **Figure** **2**; Equation S2, Supporting Information). Specifically, ≈51–71.6% of the pendent vinyl groups (i.e., the Cyclized ratios of the six polymerization systems in Table 1) underwent reaction during the linear growth phase, with about half undergoing cyclization with their own polymer backbones. This atypical consumption of vinyl groups contrasts significantly with the established role of divinyl monomers as cross‐linkers in copolymerization systems.^[^ ^27^, ^30^, ^31^, ^32^, ^33^, ^34^, ^35^ ^]^ Also, compared to our previous work^[^ ^22^ ^]^ (where the Cyclized ratio of poly(ethylene glycol dimethacrylate (EGDMA)) is below 30%), the Cyclized ratio of poly(PEGDA) in this work is much higher. This is due to the significantly lowered reaction concentration in this study ([M]_0_ = 0.1 m) compared to the previous one ([M]_0_ = 1.45 m), instead of the differences in the distance between double bonds of a monomer (a thorough discussion on how each reaction parameter impacts on the resulted cyclized structure will be reported in our future work). These findings suggest that intramolecular cyclization is a predominant process during the linear growth phase. The pendent vinyl groups were likely consumed through intramolecular reactions, integrating into their own polymer backbones to form self‐knotted chain architectures. Moreover, the evolution of pendent vinyl conversion with *Conv*. was plotted for poly(PEGDA_575_) (Figures S2 and S3, Supporting Information). It can be seen that at the very early stage (*Conv*. <10%), a high Cyclized ratio was reached, and later on, with the occurrence of both intramolecular cyclization and intermolecular cross‐linking, the pendent vinyl conversion represented the sum of Cyclized ratio and Branched ratio, which gradually increased with further *Conv*.

The initiator contents are summarized in Table 1 (Equation S3, Supporting Information). For typical branched polymers, the initiator content (the number of initiators divided by the number of PEGDA units) in the polymer product should be equal to or slightly lower than the branching unit content.^[^ ^36^ ^]^ However, in all poly(PEGDA) systems in this study, the initiator content in the polymer product is significantly lower than the PEGDA unit content. This suggests that in these high monomer‐to‐initiator ratio systems, there are numerous connections along each main chain, indicating the presence of a substantial number of “loops” in the polymer product. Moreover, the initiator content correlated well with the theoretical molecular weight (*M*_n,th_ in Table S1, Supporting Information), further demonstrating the linear single‐chain characteristics of the cyclized polymers.

**Figure 3:**
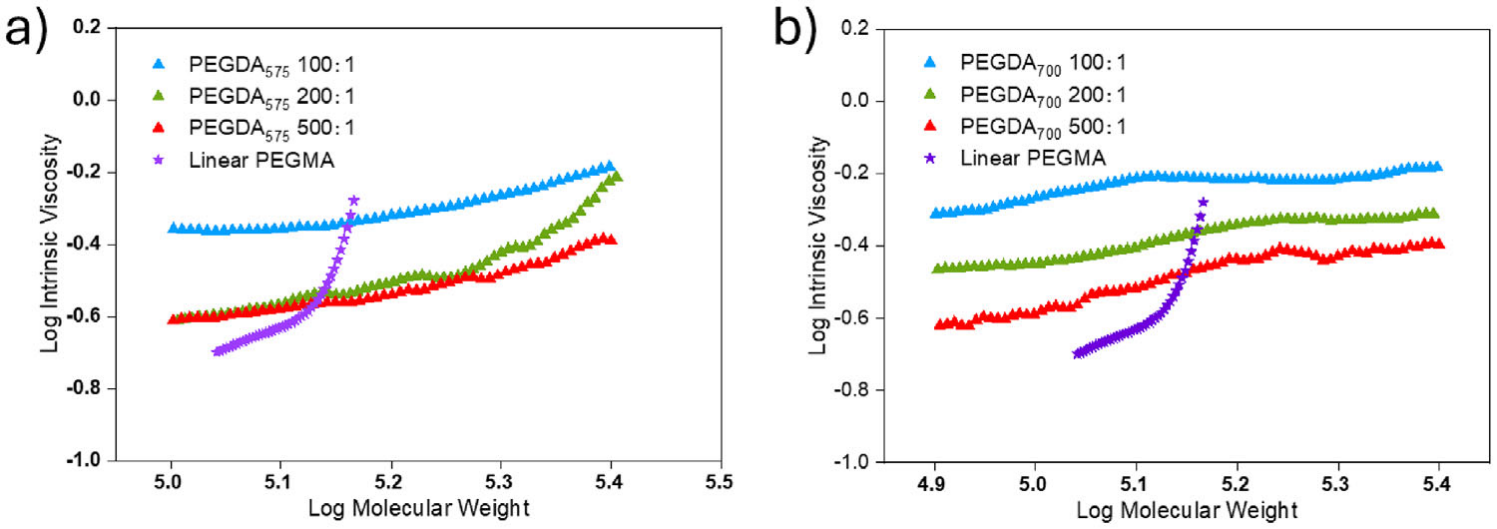
Mark–Houwink plots for the polymers obtained by homopolymerization of a) PEGDA_575_ and b) PEGDA_700_ using different monomer‐to‐initiator ratios. The Mark–Houwink plots of a linear polymer of poly(ethylene glycol) methyl ether acrylate (PEGMA, *M*_n_ = 480) synthesized by Cu(0)&Cu^II^‐mediated RDRP are presented in the figures for comparison.

The obtained single‐chain cyclized poly(PEGDA)s were also characterized by SEC with triple detectors. The Mark‐Houwink plots of the cyclized poly(PEGDA)s and their linear counterparts show that the increase in viscosity of poly(PEGDA) solutions as molecular weight increases is less pronounced compared to linear poly(PEGMA) synthesized by the same method (**Figure** **3**). The Mark‐Houwink exponents of cyclized poly(PEGDA) are significantly lower (*α* = 0.2–0.4, typical of a “hard sphere” model, Table 1), indicating a more compact spherical conformation compared to their linear analogs, which further confirms the formation of single‐chain cyclized/knotted polymers.

The PEGDA units are inherently flexible, and as the conversion increases, the polymer's flexibility improves, providing sufficient driving force for intramolecular cyclization. Interestingly, at the same monomer‐to‐initiator ratio and similar *Conv*_._, single‐chain cyclized/knotted poly(PEGDA_575_) exhibits a higher degree of cyclization (≈71.6% for 500:1) compared to single‐chain cyclized/knotted poly(PEGDA_700_) (≈60.3% for 500:1). This is likely due to the shorter PEG chains in PEGDA_575_, which result in a higher local concentration of acrylate vinyl groups on the side chains, thereby facilitating more intramolecular reactions. Additionally, a significant number of unreacted pendent vinyl groups remain in the single‐chain poly(PEGDA) (Figure 2), which could be further functionalized using thiol‐ene click chemistry, enabling the formation of a variety of multifunctional single‐chain cyclized/knotted polymers.

**Figure 4:**
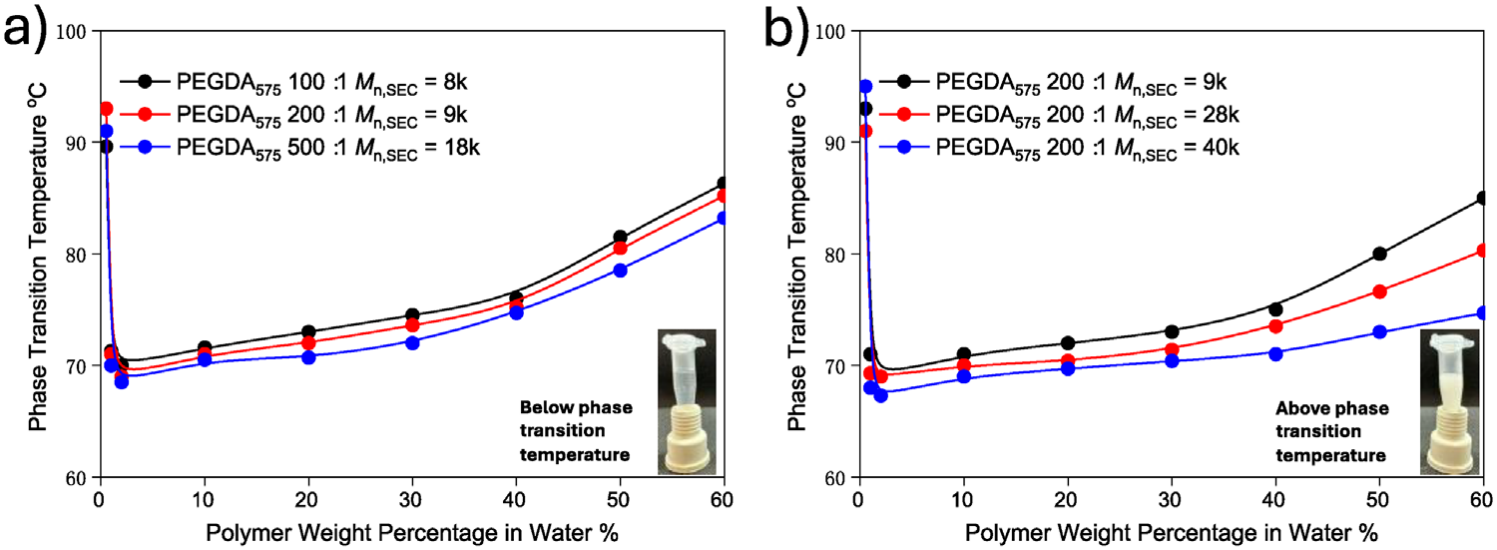
Thermoresponsive properties of the obtained poly(PEGDA_575_)s. a) phase transition temperature of the poly(PEGDA_575_)s in distilled water at different concentrations. b) Comparison of phase transition temperature of the poly(PEGDA_575_)s 200:1 obtained at different times of polymerization with different molecular weight.

As known, both poly(PEGDA_575_) and poly(PEGDA_700_) dissolve in polar solvents and many organic solvents (e.g., methanol, THF and chloroform). The cyclized product derived from PEGDA_700_ (with *M*_n,NMR_ of 11, 20, and 45 kDa) shows excellent solubility in aqueous solutions regardless of molecular weight due to the longer PEG chains providing enhanced hydrophilicity. However, the product obtained from PEGDA_575_ exhibits distinct thermoresponsive behavior in water (**Figure** **4**).

Specifically, when the temperature reaches a certain threshold, the polymer produced from PEGDA_575_ precipitates from the solution, resulting in phase separation. Numerous studies have explored the phase transition temperature of PEG‐based polymers at low concentrations (typically 0.2% w/v), and more recently, the phase transition temperature of hyperbranched PEGDA polymers has also been examined.^[^ ^21^ ^]^ However, the phase transition temperature of cyclized PEGDA polymers has not yet been reported.

As illustrated in Figure 4a, when the polymer concentration is ≈2% w/v, poly(PEGDA_575_) samples with different monomer‐to‐initiator ratios (100:1, 200:1, and 500:1) exhibit lower critical solution temperatures (LCSTs). The LCST values for poly(PEGDA_575_) at ratios of 100:1, 200:1, and 500:1 are 70, 69, and 68.5 °C, respectively. Additionally, we observed that the phase transition temperature of poly(PEGDA_575_)s exhibits a strong dependence on polymer concentration. Notably, the phase transition temperature for all three poly(PEGDA_575_) samples followed a similar trend and rate of change as the polymer concentration increased. Specifically, the LCST of poly(PEGDA_575_)s increased by ≈15 °C when the polymer concentration was raised from 2% to 60% w/v. Based on the chemical structure of cyclized poly(PEGDA_575_), the hydrophobic components of the polymer are the initiator end groups and the polymer backbone, while the PEG chains are hydrophilic. In general, a higher number of PEGDA units enhances hydrogen bonding interactions between the polymer and water, thereby broadening the miscibility temperature range. In contrast, a higher number of initiator end groups or a longer backbone increases the thermodynamic cost of solvation, which lowers the LCST. It is worth noting that, as the length of the polymer backbone increases, the number of PEG chains also increases, making the impact of the backbone less significant compared to that of the initiator. However, as shown in Figure 4a, the LCST values for poly(PEGDA_575_) at three different initiator contents are generally similar. It is well known that the phase transition temperature of a polymer depends on various factors affecting its solubility. Therefore, there must be other factors that have an effect on the LCST opposite to that of the initiator content mentioned above, thereby offsetting the impact of each other. According to previous studies, an increase in molecular weight tends to lower the phase transition temperature and broaden the transition curve (phase transition lines) due to the increased energy cost of solvation.^[^ ^21^, ^37^, ^38^ ^]^ Indeed, this trend is clearly demonstrated in Figure 4b, where the phase transition behavior of poly(PEGDA_575_) at a 200:1 monomer‐to‐initiator ratio with different molecular weights is examined. As seen in Figure 4b, as the molecular weight increases, the phase transition temperature at each polymer concentration decreases, and the curves of transition temperature – polymer weight percentage in water broadens (as *M*_n,SEC_ increases from 9 to 40 kDa), the LCST trends become less steep across the polymer weight percentages. This flattening of the curves indicates broader thermal transitions as the molecular weight increases, particularly at higher polymer concentrations. Specifically, the 9 kDa poly(PEGDA_575_) increased by 16 °C (from 69 to 85 °C), the 28 kDa increased by 11.3 °C (from 69 to 80.3 °C), while the 40 kDa increased by only 7.4 °C (from 67.3 to 74.7 °C). However, the observed LCST differences in Figure 4b are not as significant as expected. This is primarily related to the unique structure characteristics of the PEG‐based cyclized polymers. Typically, an increase in molecular weight would reduce solubility, resulting in a lower LCST. However, for the PEG‐based cyclized polymers here, the increase in molecular weight has two opposing effects: on the one hand, it introduces more hydrophilic PEG chains; on the other hand, it leads to a more compact knotted architecture, which might encapsulate the hydrophobic polymer backbone. Both structural factors counteract the usual effect of molecular weight on LCST, causing the LCST‐lowering effect of molecular weight to appear less pronounced in this case.

Moreover, compared to the LCST of hyperbranched polymers (e.g. 30–40 °C) reported previously (with *M*_n,SEC_ of 39 kDa),^[^ ^21^ ^]^ the LCST of the single‐chain cyclized/knotted polymers in this study (with *M*_n,SEC_ of 40 kDa) is significantly higher, reaching 70–90 °C, indicating a significant impact of the cyclized structure on the polymer's solubility (no LCST was observed for the water‐soluble linear PEGMA polymers, Figures S4 and S5, Supporting Information). The highly cyclized structure notably elevates the LCST, which may be attributed to the lower overall initiator concentration in the knotted polymers, leading to increased hydrophilicity compared to hyperbranched polymers. A more plausible explanation lies in the compact structure of cyclized polymers, which can shield hydrophobic groups within their cores. This structural feature minimizes the exposure of hydrophobic units to water, allowing the outer regions of the polymer to interact more favorably with water molecules, thereby increasing the LCST. In contrast, hyperbranched polymers, with their more open and flexible architectures, expose more hydrophobic groups, resulting in a lower LCST. This relatively high LCST of cyclized polymers compared to the hyperbranched ones makes them more suitable for applications in physical conditions, with various molecular weights. As the focus of this current work is to, for the first time, report the LCST phenomenon of this newly synthesized water‐soluble single cyclized/knotted polymer, a systematic study on the relationship of polymer structure (linear, branched, cyclized) and their thermoresponsive properties will be reported in future work.

## 3 Conclusion 

A series of water‐soluble single‐chain cyclized/knotted polymers were synthesized through the homopolymerization of PEGDA for the first time, facilitated by enhanced intramolecular cyclization. The formation of the single‐chain cyclized/knotted polymer structure was achieved by maintaining a high monomer‐to‐initiator ratio and strong deactivation strength, resulting in a low chain concentration and increasing the likelihood of intramolecular cyclization before cross‐linking could occur. Furthermore, Both poly(PEGDA_575_)s and Poly(PEGDA_700_)s demonstrated excellent solubility in aqueous solutions, whereas cyclized/knotted poly(PEGDA_575_)s exhibited distinct thermoresponsive behavior at relatively high temperatures (≈70–90 °C), which was influenced by initiator content, molecular weight and polymer concentration. These water‐soluble single‐chain cyclized/knotted polymers contain numerous pendent vinyl groups, offering great versatility for post‐functionalization. Moreover, due to the relatively high LCST of cyclized/knot polymers (much higher than body temperature), they can post‐cross‐link with functionalized biopolymers to fabricate novel hydrogels for various biomedical applications.

## Conflict of Interest

The authors declare no conflict of interest.

## Author Contributions

Z.L. conducted the experiments and drafted the manuscript; Z.L. and J.L. contributed to the data analysis; Y.L., R.S., C.Z., M.J., and T.M. carried out portions of the experimental work; H.T. contributed to manuscript editing; J.L. and W.W. reviewed and revised the manuscript; J.L. proposed the concept; W.W. provided funding support.

## Ethical Statement

This study does not involve human participants or animal subjects

## Supporting information



Supporting Information

## Data Availability

The data that support the findings of this study are available from the corresponding author upon reasonable request.
