# Supplementary material for: Water Soluble Thermoresponsive Single‐Chain Cyclized/Knotted Polymers from Intramolecular Cyclization Dominated Homopolymerization of PEG Diacrylate
Source: Macromol Rapid Commun. 2025 Mar 10;46(12):2401068. doi: 10.1002/marc.202401068 (PMC12183152; doi:10.1002/marc.202401068)
Supplement: Supplementary file 1 — Supporting Information [file MARC-46-2401068-s001.docx]

**Supporting Information**

**Water soluble thermoresponsive single-chain cyclized/knotted polymers from intramolecular cyclization dominated homopolymerization of PEG diacrylate**

*Zishan Li^1^, Jing Lyu^*,1^, Yinghao Li^1^, Rijian Song^1^, Chunyu Zhao^1^,* *Melissa Johnson^1^, Tianyu Mao^2^, Hongyun Tai^3^, Wenxin Wang^*,1,2^*

^1^ Charles Institute of Dermatology, School of Medicine, University College Dublin, Dublin 4, Ireland

^2^ School of Mechanical and Materials Engineering, University College Dublin, Dublin 4, Ireland

^3^ Blafar Limited, Cherrywood Business Park, Cherrywood, Loughlinstown, Dublin 18, Ireland

*Corresponding author (email: jing.lyu@ucd.ie, wenxin.wang@ucd.ie)

**EXPERIMENTAL SECTION**

**Materials**

Poly(ethylene glycol) diacrylate (PEGDA_575_ and PEGDA_700_, 98% Sigma-Aldrich), ethyl α-bromoisobutyrate (EBriB, 98%, Aldrich), pentamethyldiethylenetriamine (PMDETA, 99%, Aldrich), copper(II) bromide (CuBr_2_, 99%, Aldrich), d-chloroform (99.8%, Aldrich), were used as received unless otherwise stated. Copper wire was purchased from Sigma-Aldrich, then immersed in conc. HCl and rinsed with water and acetone before use. Dimethyl sulfoxide (DMSO) and diethyl ether (Fisher Scientific) and Al_2_O_3_ (basic, Fisher) were used as received.

**Synthesis of single-chain cyclized/knotted poly(PEGDA)**

The polymerizations were carried out in a two-necked round-bottom flask, with the ratios of monomer and initiator set as 100/1, 200/1 and 500/1 for both PEGDA_575_ and PEGDA_700_. Taking the system of PEGDA_575_ or PEGDA_700_ with the ratio of 100/1 as an example, PEGDA_575_ (8.63 g, 15 mmol, 100 equiv.) or PEGDA_700_ (10.50 g, 15 mmol, 100 equiv.), EBriB (29.26 mg, 0.15 mmol, 1 equiv.), PMDETA (25.06 μL, 0.12 mmol, 0.8 equiv.), CuBr_2_ (13.40 mg, 0.06 mmol, 0.4 equiv.) and DMSO (142.25 mL for PEGDA_575_, 140.58 mL for PEGDA_700_) were added into the two-neck flask and bubbled with argon to remove oxygen for 15 min. A stirrer bar wrapped with 5 cm of copper wire was immersed in conc. HCl and then thoroughly rinsed with acetone and water. After the pre-treated Cu(0) wire was dried, it was added to the flask quickly under a positive pressure of argon. The reaction was stirred at 600 r/min in an oil bath at 25 °C, and polymerization was conducted for a desired period of reaction time. For the system of PEGDA_575_ and PEGDA_700_ with the ratio of 200/1 and 500/1, the same polymerization procedures were used, except that different amount of EBriB was used (14.63 mg, 0.08 mmol, 0.5 equiv. for 200/1; 5.85 mg, 0.03 mmol, 0.2 equiv. for 500/1). The experiment was terminated by opening the reaction flask and allowing the reaction to be exposed to air. The resulting solution was subsequently diluted with DMSO and precipitated into an excess amount of cold diethyl ether to remove any unreacted monomers. The precipitate was dried under laminar flow and then re-dissolved in acetone, followed by three passes through an Al_2_O_3_ column for purification. Finally, the mixture was dried under vacuum.

**Synthesis of linear polymer of poly(ethylene glycol) methyl ether acrylate (PEGMA, *M*_n_ = 480)**

The polymerizations were carried out in a two-necked round-bottom flask. PEGMA (7.20 g, 15 mmol, 100 equiv.), EBriB (22.01 mg, 0.15 mmol, 1 equiv.), PMDETA (25.06 μL, 0.12 mmol, 0.8 equiv.), CuBr_2_ (13.40 mg, 0.06 mmol, 0.4 equiv.) and DMSO (143.35 mL) were added into the two-neck flask and bubbled with argon to remove oxygen for 15 min. A stirrer bar wrapped with 5 cm of copper wire was immersed in conc. HCl and then thoroughly rinsed with acetone and water. After the pre-treated Cu(0) wire was dried, it was added to the flask quickly under a positive pressure of argon. The reaction was stirred at 600 r/min in an oil bath at 25 °C, and polymerization was conducted for a desired period of reaction time. The experiment was terminated by opening the reaction flask and allowing the reaction to be exposed to air. The resulting solution was subsequently diluted with DMSO and precipitated into an excess amount of cold diethyl ether to remove any unreacted monomers. The precipitate was dried under laminar flow and then re-dissolved in acetone, followed by three passes through an Al_2_O_3_ column for purification. Finally, the mixture was dried under vacuum.

**Size exclusion chromatography (SEC)**

At predetermined time intervals, samples were extracted from the reaction mixture using a glass syringe equipped with a Luer needle under positive argon pressure. The collected aliquots were subsequently diluted in dimethylformamide (DMF), passed through an Al_2_O_3_ pipette for purification, and then filtered using a 0.4 μm filter before undergoing chromatographic analysis. Number-average molecular weight (*M*_n,SEC_), weight-average molecular weight (*M*_w,SEC_) and dispersity (*Đ*), *Conv*_._, Mark–Houwink exponent *α* were obtained by size exclusion chromatography (SEC) (Agilent GPC/SEC 50) equipped with a refractive index (RI) and light scattering (LS) detectors. The columns were eluted using DMF (30 cm PLgel Mixed-C * 2) and calibrated using a series of 12 near-monodisperse PMMA standards (*M*_p_ from 690 g/mol to 1.944 × 10^6^ g/mol). The methods were set at a rate of 1 mL/min at 40℃.

**Nuclear magnetic resonance (NMR) spectroscopy**

The polymers were dissolved in CDCl_3_ for ^1^H NMR analysis. ^1^H NMR analysis was carried out on a Varian NMR system 400 MHz spectrometer with MestRenova 6.1 processing software and reported in parts per million (ppm) relative to the response of the CDCl_3_ (7.26 ppm) and tetramethylsilane (0.00 ppm).

**Phase transition temperature measurement**

The phase transition temperatures were measured in aqueous polymer solutions using turbidity analysis with a UV-vis spectrometer equipped with temperature control. Light transmittance at 500 nm was recorded for polymer solutions at different concentrations. The phase transition temperature was identified as the point where the transmittance reached 90% during heating.

**Table S1.** SEC data of both PEGDA_575_ and PEGDA_700_ at different monomer-to-initiator ratios and time points during the polymerization

| Entry | Diacrylate | M: I*^a^* | Time (h) | *﻿ Conv^b^* (%) | *M*_n,th_*^c^* (kDa) | *M*_n,SEC_*^d^* (kDa) | *M*_w,SEC_*^d^* (kDa) | *Đ^d^* |
| --- | --- | --- | --- | --- | --- | --- | --- | --- |
| 1 | PEGDA_700_ | 100:1 | 1 | 5.8 | 4.06 | 4.75 | 5.60 | 1.18 |
| 2 |  |  | 2.5 | 17.4 | 12.18 | 7.57 | 10.46 | 1.32 |
| 3 |  |  | 4 | 29.5 | 20.6 | 12.6 | 19.70 | 1.57 |
| 4 |  |  | 6.5 | 43.4 | 30.38 | 25.06 | 49.44 | 1.97 |
|  |  |  | 10 | 59.7 | 41.79 | 55.23 | 177.83 | 3.38 |
| 5 |  | 200:1 | 3 | 10.1 | 14.14 | 7.99 | 9.25 | 1.16 |
| 6 |  |  | 4 | 16.4 | 22.96 | 11.14 | 13.47 | 1.21 |
| 7 |  |  | 6.5 | 29.1 | 40.74 | 20.71 | 28.96 | 1.40 |
| 8 |  |  | 8 | 36.5 | 51.10 | 30.44 | 49.07 | 1.61 |
|  |  |  | 16 | 56.5 | 79.10 | 94.92 | 296.98 | 3.13 |
| 9 |  | 500:1 | 4 | 6.0 | 21.00 | 12.20 | 13.71 | 1.12 |
| 10 |  |  | 6 | 11.4 | 39.90 | 22.04 | 25.67 | 1.17 |
| 11 |  |  | 8 | 18.1 | 63.35 | 35.49 | 47.20 | 1.33 |
| 12 |  |  | 10 | 25.7 | 89.95 | 53.96 | 84.29 | 1.56 |
|  |  |  | 22 | 51.3 | 179.55 | 255.67 | 1301.78 | 5.09 |
| 13 | PEGDA_575_ | 100:1 | 1 | 5.1 | 2.93 | 3.67 | 4.13 | 1.12 |
|  |  |  | 2.5 | 16.1 | 9.26 | 6.65 | 7.98 | 1.20 |
| 14 |  |  | 4 | 30.1 | 17.31 | 11.22 | 14.7 | 1.30 |
| 15 |  |  | 5.5 | 42.8 | 24.61 | 18.53 | 27.03 | 1.46 |
| 16 |  |  | 7.5 | 56.7 | 32.60 | 31.15 | 57.31 | 1.84 |
|  |  |  | 9h | 65.9 | 37.89 | 47.61 | 107.38 | 2.26 |
| 17 |  | 200:1 | 2 | 7.6 | 8.74 | 4.89 | 5.57 | 1.12 |
|  |  |  | 3 | 13.5 | 15.53 | 7.69 | 9.00 | 1.17 |
| 18 |  |  | 6.5 | 38.6 | 44.39 | 24.93 | 36.33 | 1.46 |
| 19 |  |  | 8 | 49.8 | 57.27 | 36.36 | 64.05 | 1.76 |
| 20 |  |  | 10 | 58.5 | 67.27 | 54.65 | 119.5 | 2.19 |
|  |  |  | 14 | 78.1 | 89.81 | 160.25 | 977.17 | 6.10 |
| 21 |  | 500:1 | 3 | 5.5 | 15.81 | 7.40 | 8.36 | 1.13 |
|  |  |  | 5 | 10.7 | 30.76 | 15.13 | 17.15 | 1.14 |
| 22 |  |  | 8.5 | 25.2 | 72.45 | 37.53 | 51.81 | 1.38 |
| 23 |  |  | 9.5 | 28.4 | 81.65 | 43.76 | 64.14 | 1.47 |
| 24 |  |  | 12.5 | 46.4 | 133.40 | 101.65 | 218.03 | 2.14 |
|  |  |  | 19 | 61.5 | 176.81 | 195.66 | 846.50 | 4.33 |

*^a^*[M]_0_/[I]_0_/[Cu^II^]_0_/[L]_0_ = 100/[I]_0_(=1; 0.5; 0.2)/0.4/0.8; M: polyethylene glycol diacrylate; I: ethyl α-bromoisobutyrate (EBriB), Cu^0^ = pretreated Cu(0) wire (l = 5 cm, d = 1 mm); Cu^II^ = CuBr_2_, L = PMDETA; solvent = DMSO. *^b^*The monomer conversion is calculated by comparing the integrated areas of the polymer and monomer peaks in the SEC trace. *^c^M*_n,th_ = ([M]_0_/[I]_0_) × Monomer conversion (*Conv*_._) × MW (M). *^d^M*_n,SEC_, *M*_w,SEC_ and *Đ* are determined by SEC using PMMA as standards in DMF.

**Determination of the polymer composition and branching ratio**

^1^H NMR analysis for the poly(PEGDA)s demonstrates the presence of a high amount of vinyl functional groups at characteristic peaks between 6.5 ppm and 5.7 ppm, as shown in Figure 2. The poly(PEGDA)s composition was determined by integrating a, d, d’ and k,i peaks (Figure 2). Eq. S1 - S3 outline the calculations:

Vinyl content (mol %) $\text{=}\frac{\text{a}}{\text{[}\text{d+d'-(k+i)/3}\text{]}\text{/}\text{4}}$

S1

Pendent vinyl conversion or Cyclized ratio (mol %) $\text{=}\text{1}-\frac{\text{a}}{\text{[}\text{d+d'-(k+i)/3}\text{]}\text{/}\text{4}}$

S2

Initiator content (mol %) $\text{=}\frac{\text{(}\text{k+i)}\text{/9}}{\text{[}\text{d+d'-(k+i)/3}\text{]}\text{/}\text{4}}$

S3

*M*_n,NMR_ (g/mol) $\text{=}$ $\frac{1}{Initiator content (mol \%)}\times(700 or 575 g/mol)$

S4


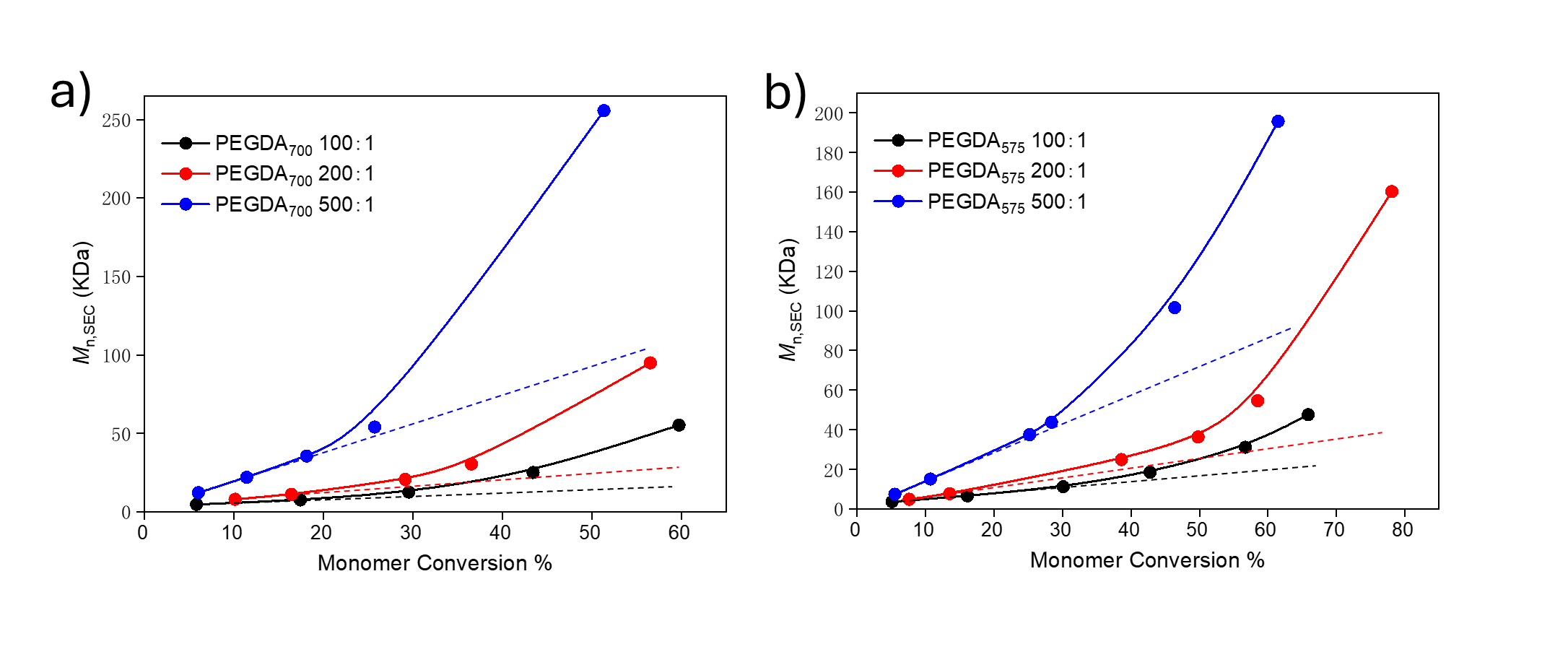


**Figure S1.** Dependence of the *M*_n,SEC_ on the monomer conversion for the homopolymerization of PEGDA_575_ and PEGDA_700_ *via* Cu(0)&Cu^II^-mediated RDRP.

**Figure S2.** ^1^H NMR spectra of poly(PEGDA_575_) with [M]_0_/[I]_0_ of 200/1 at different monomer conversion.

**Figure S3.** The kinetic plot of pendent vinyl conversion as a function of monomer conversion for poly(PEGDA_575_) with [M]_0_/[I]_0_ of 200/1.

**
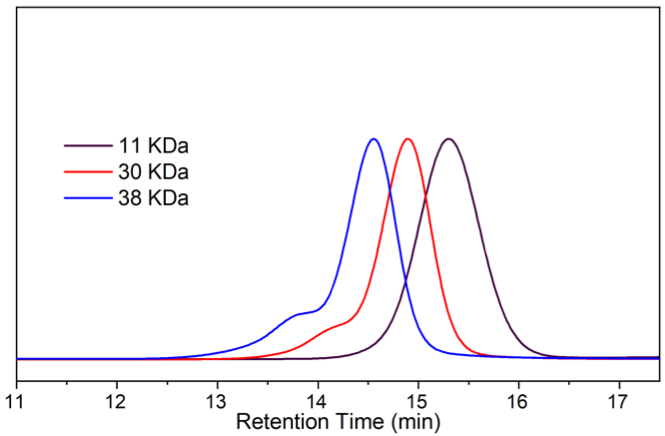
**

**Figure S4.** SEC traces of poly(PEGMA) with molecular weights of 11 kDa, 30 kDa and 38 kDa.

**
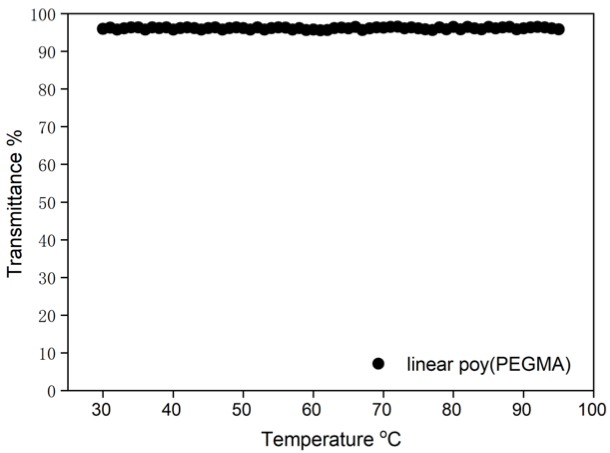
**

**Figure S5.** Plot of transmittance as a function of temperature for linear poly(PEGMA) with molecular weights of 38 kDa (polymer weight percentage in water 30%).
